# Flexible and Optical Fiber Sensors Composited by Graphene and PDMS for Motion Detection

**DOI:** 10.3390/polym11091433

**Published:** 2019-08-31

**Authors:** Dong Wang, Bin Sheng, Lina Peng, Yuanshen Huang, Zhengji Ni

**Affiliations:** 1 School of Optical Electrical and Computer Engineering, University of Shanghai for Science and Technology, Shanghai 200093, China; 2 Shanghai Key Laboratory of Modern Optical Systems, Engineering Research Center of Optical Instruments and Systems, Shanghai 200093, China; 3 Shanghai Institute of Optical Instruments, Shanghai 200093, China

**Keywords:** flexible sensor, optical sensor, optical loss, graphene, PDMS, composite fiber, motion detection

## Abstract

A stretchable optical sensor can quantify the strain generated by human movement, which has been widely studied in the development of health monitoring systems, human–machine interfaces and wearable devices. This paper reports a graphene-added polydimethylsiloxane (PDMS) fiber, which has high tensile properties and good light transmittance suitable for detecting human movement. When the graphene-added PDMS fiber is stretched, the concentration of graphene per unit volume is constant, and the sensor uses the optical loss of the beam through the graphene PDMS fiber to detect the tensile strain. The fiber has excellent strain-sensing performance, outstanding sensitivity, a tensile property of 150%, and an excellent waterproofing performance. The linear response and repeated response in large dynamic range could reach 100% stability. The results show that the sensor can be used to detect human motion detection. These excellent properties indicate that the fiber has potential applications in wearable devices, soft robots and electronic skin.

## 1. Introduction

In recent years, flexible, stretchable and wearable sensor devices have received widespread attention [1,2,3,4,5,6,7,8,9]. Because the elastic materials have low-cost, scalable, and simple production methods, elastic strain sensors are widely used in a variety of practical applications, such as artificial electronic skins [10,11,12,13], motion detection [14,15], and soft robots [16]. In quantitative detection of strain in flexible materials, strain sensors are required to be sufficiently sensitive to be mechanically compatible and capable of withstanding large deformations. As an imitation of human motion perception ability, the flexible and stretchable strain sensor is expected to achieve the perception performance of human joint movement. The stretchable strain sensors should be able to deform and closely adhere to any surface to achieve accurate monitoring of the signal.

In order to meet these needs, many studies have reported flexible, stretchable and highly sensitive strain or pressure sensors made of graphene polymer [17,18,19,20], carbon nanotubes [21,22] and nanowire composite structures [23,24,25]. For flexible electronic sensors, the detection of strain is usually based on the change by capacitance or resistance under mechanical variability, and the sensitivity of the strain sensor is reflected by the rate of change. Currently, the miniaturization of electronic sensors, the current leakage caused by insufficient insulation and the high sensitivity to electromagnetic interference are still difficult and challenging practical problems. In contrast, optical sensors provide an excellent solution to these challenges, especially flexible fiber optic sensors. Compared to flexible electronic sensors, flexible fiber optic sensors are resistant to electromagnetic interference and safe. So far, some encouraging developments have been made in flexible fiber optic sensing applications. Among them, Yang et al. [26] pioneered a gel fiber that can carry up to 700% strain and can be implanted. The publication of this paper, considering the environment as well as the material, enhanced the performance of many follow-up studies. In 2017, Yang et al. [27] reported a fiber optic sensor for human motion detection. The tensile strain was measured by dye-doped PDMS, the absorption characteristics of the dye molecules were measured by absorption spectroscopy, and a highly sensitive response was obtained. Fang et al. [28] proposed and demonstrated a novel flexible and elastic vibration-displacement fiber sensor with a doped polydimethylsiloxane (PDMS) micro-fiber based on model interference. Therefore, the composite materials formed by the combination of organic and inorganic materials are a reliable method to improve the sensitivity of strain sensors.

This paper reports a graphene-added PDMS fiber with high tensile properties and good light transmittance. We studied the beam attenuation after passing through the graphene-added PDMS fiber to detect tensile strain. The optical loss is mainly caused by the absorption and scattering of graphene. The sensor can easily reach 150% strain, which shows the large range of human joint detection capabilities, including finger bending, wrist bending, elbow bending and knee bending. The muscle detection of human movement also exhibits excellent sensitivity including arm muscle relaxation and tension, the exhalation and inhalation of the human body. In addition, we demonstrated waterproof properties and tested the sensor changes in different environments. According to the results of this research, it is expected that people will be able to cost-effectively manufacture sensors that meet a variety of human needs.

## 2. Materials and Methods 

### 2.1. Materials

Single-layer graphene (diameter about 0.2–10 μm; thickness about 1 nm) with a purity of over 99% was acquired from Suzhou Tanfeng Materials Tech. Co. Ltd. (Suzhou, China) and was prepared by a chemical method. Poly tetra fluoroethylene (PTFE) tubes with different diameter sizes were bought from Oudelixin (Shanghai, China). Polydimethylsiloxane (PDMS, Sylgard 184) was supplied by Dow Corning Co. Ltd. (Michigan, USA) The medical rubber gloves and the injection syringe were all purchased from a local hospital (Shanghai, China).

### 2.2. Fabrication Procedures of Optical Fiber 

**Figure 1:**
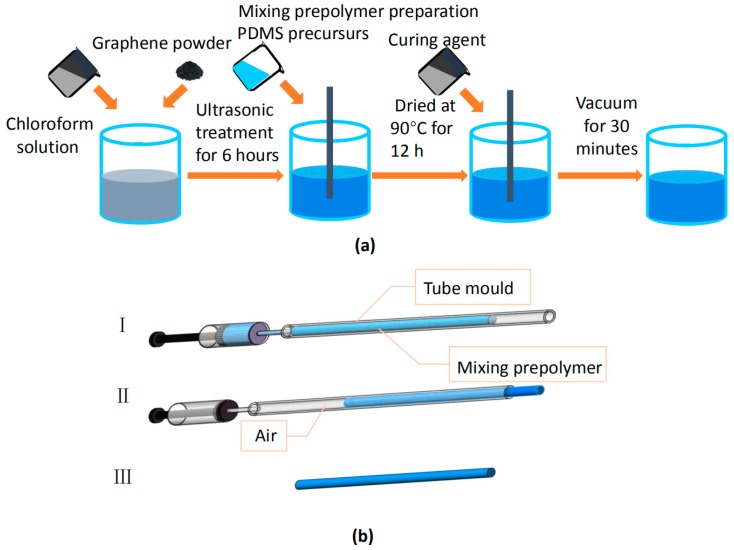
(**a**) Schematic showing the preparation of the mixture prepolymer. (**b**) PDMS fiber preparation process. (I) Extract the prepared PDMS mixture and inject into a silicone tube mold, cure in the natural state for 24 hours; (II) extrude the PDMS fiber by air pressure; (III) optical fiber schematic of PDMS fiber.

A simple two-step method was used to prepare the graphene-added PDMS fiber. Firstly, the graphene powder was dispersed (5 mg/ml) in chloroform (Samchun, Seoul, Korea). After ultrasonic treatment for 6 hours, a well-dispersed solution was mixed with polydimethylsiloxane. Subsequently, the mixing solute on was mixed with a vortex and dried at 90 °C for 12 h in order to completely evaporate the chloroform. The mixing prepolymer was prepared by polydimethylsiloxane and a curing agent at a ratio of 10:1 (Sylgard 184, Dow Corning) by weight. The uniformly stirred PDMS mixture was degassed in a vacuum box. Figure 1a shows the entire preparation process. The concentration of graphene is the percentage of graphene in the mixing prepolymer per unit mass. Secondly, for the preparation of the graphene-added PDMS fiber, the degassed PDMS precursor was injected into a silicone tube mold (inner diameter of 1 mm) with a syringe and placed in a vacuum drying, as shown in Figure 1b. Once the liquid components were thoroughly mixed, the mixture were cured to a flexible elastomer. The curing process is gradual, depending on the temperature, and associated with an increasing viscosity of the elastomer. According to the manufacturer, the complete curing phase occurs after 24 hours at 25 °C. The diameter of the fiber was determined by the inner diameter of the tube mold. After curing, the fiber was drawn out of the mold using air pressure. 

### 2.3. Optical Fiber Measurement 

The High Power UV–Vis Fiber Light Source (L10290, Hamamatsu Photonics, Hamamatsu, Japan), which outputs 200 nm to 1600 nm light through a light guide, was used as the light source. The emission wavelength was 441.6 nm helium-cadmium laser. The sensor was coupled with two UV resistant quartz fibers (core: 600 μm) at each end, encapsulated with fixture equipment. Digital light power and an energy meter (PM100D) were used to measure the light intensity variation of the fiber optic sensor. The Laser Star Dual Channel (P/N 7Z01601) is a laser power meter, which is driven by window software. An Ocean Optics USB4000 spectrometer (Florida, USA) measured the transmission spectrum and the spectral range was 200–1100 nm.

## 3. Results

### 3.1. Characteristics of Fiber Optic Sensors

**Figure 2:**
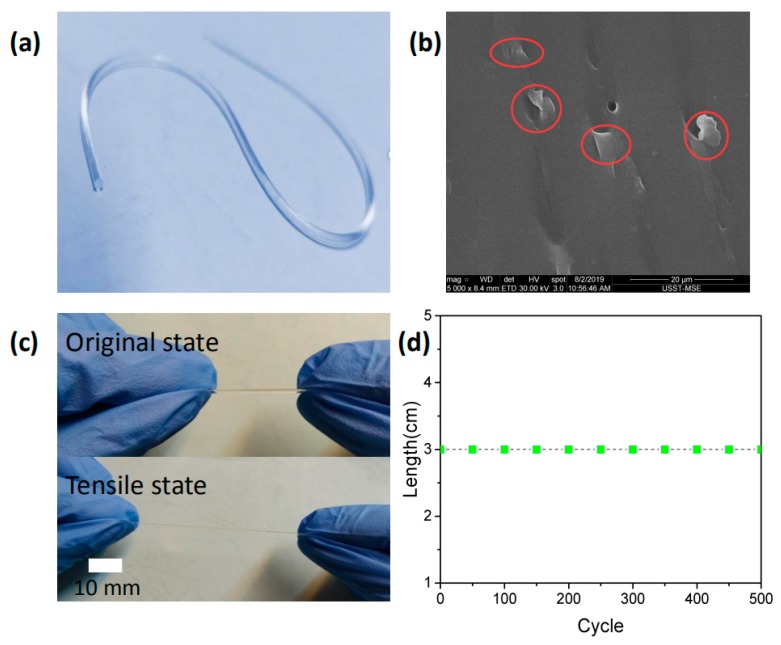
(**a**) Digital optical image of graphene-added PDMS fiber. (**b**) SEM image of PDMS fiber cross-section. The concentration of the tested sample is *c* = $5\times 10^{-4}\;\mathrm{wt}\;\%$ and the red marks are graphene. (**c**) Digital optical images of the PDMS fiber under the original (top) and stretched (bottom) state, showing the excellent stretchability of the PDMS fiber. (**d**) The length of the same PDMS fiber was measured and repeated circulation every 100 times after 100% strain.

We investigated the stretchability of the graphene-added PDMS fiber with a length of 3 cm and a diameter of 1 mm. The digital optical of graphene-added PDMS fiber is shown in Figure 2a. We found some signs that graphene might exist, the results consistent with the actual graphene size (diameter 0.2–10 μm), as shown in Figure 2b. The concentration of the tested sample is *c* = $5\times 10^{-4}\;\mathrm{wt}\;\%$ and the red marks are graphene. The maximum tensile capacity of graphene-added PDMS fiber can reach 150%, as shown in Figure 2c. To evaluate the mechanical durability, we stretched the PDMS fiber with length of 3 cm and diameter of 1 mm to 100% strain and measured the fiber length every 100 cycles, as shown in Figure 2d. Even if the stretching cycle was repeated 500 times, the length of the PDMS fiber did not change.

### 3.2. Sensing Mechanism of Fiber Optic Sensors

**Figure 3:**
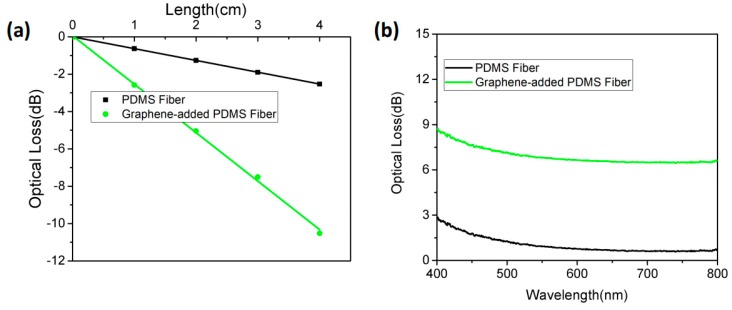
The concentration of the tested graphene-added PDMS fiber is c = $5\times 10^{-4}\;\mathrm{wt}\;\%$. (**a**) The function of optical loss and fiber length. (**b**) Optical attenuation spectra of the fiber.

In order to detect the optical properties of PDMS fibers, the optical properties of graphene-added PDMS materials were characterized by optical attenuation spectroscopy. We measured the transmission loss of PDMS fiber and graphene-added PDMS fibers in air, as shown in Figure 3a. A laser of 441.6 nm was emitted from one end of the fiber and the transmitted light power of the fiber with different lengths was measured, and then the light attenuation was calculated. 

As shown in Figure 3a, the optical loss of the fiber increased linearly with the transmission length, where the optical loss coefficient of PDMS was 0.63 dB/cm, and the optical loss coefficient of the graphene-added one was 2.58 dB/cm. The concentration of the tested graphene-added PDMS fiber is *c* = $5\times 10^{-4}\;\mathrm{wt}\;\%$. Evidently, the PDMS fiber with graphene-added had a larger loss absorption coefficient and a significantly higher sensitivity than the PDMS fiber in sensor applications. Figure 3b shows the optical attenuation spectra of the PDMS fiber and graphene-added PDMS fiber in the wavelength range of 400–800 nm with a length of 3 cm. The graphene-added PDMS fiber had more optical loss than PDMS fiber. In addition, we found that graphene-added PDMS fiber had no wavelength selectivity for visible wavelengths [29]. The increased attenuation of graphene-added PDMS fiber was due to the absorption and scattering of graphene randomly distributed in the doped PDMS fiber space. Therefore, the sensing mechanism of the optical sensor was based on the spectroscopy of the graphene attenuation change in the transmitted light intensity.

**Figure 4:**
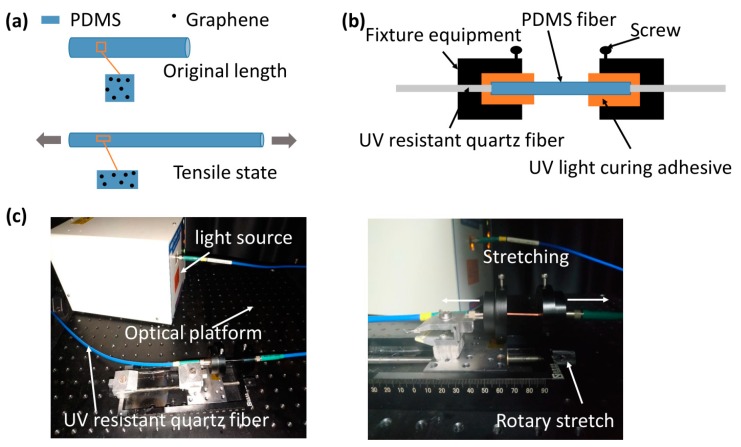
(**a**) Sensing mechanism. When the fiber is stretched, the concentration of graphene per unit volume remains the same. (**b**) Scheme diagram of the fixation of PDMS fiber structure. (**c**) Optical setup for the strain test.

The absorption of graphene was linearly proportional to the length of the light passing through the PDMS fiber, as shown in Figure 3a, following Beer-Lambert’s law [30]:

(1) $$A=kcl=\mathrm{lg}\left(\frac{1}{T}\right),$$

where *A* represents the absorbance, *k* is the molar absorption coefficient, and the absorption property of graphene does not rely on the incident wavelength *λ* of visible light. $l=l_{0}\left(1+\varepsilon \right)$ indicates the length of the graphene-added PDMS fiber, $l_{0}$ is the original length, and *ε* is the strain, *c* is the concentration of graphene, and, under strain conditions, as, due to the constant volume, with absorption per unit length unchanged, *c* remains constant. *T* is the transmittance. The absorption of graphene leads to the attenuation of transmitted light intensity, and the change in strain attenuation can be expressed as:

(2) $$D\left(\varepsilon \right)=kcl_{0}\varepsilon +\alpha (\varepsilon ),$$

where α(ε) indicates the light coupling loss. According to Figure 4a, when the fiber is stretched, the concentration of graphene per unit volume remains the same. So, when the concentration *c* is constant, the optical loss is proportional to the strain ε; when the strain ε is constant, the optical loss and the concentration *c* are linearly proportional.

The sensitivity of the sensor response is affected by the concentration of graphene and the length of the PDMS fiber. In our experimental test, the details of fiber fixation were as shown in Figure 4. Figure 4b shows the fixation of PDMS fiber structure, where a short length of PDMS fiber was pigtailed with two UV resistant quartz fibers (core: 600 μm) at each end and encapsulated with fixture equipment. UV light curing adhesive was used to reinforce the connection joint. The experimental device is shown in Figure 4c. The PDMS fiber was glued on the circular holes of two pieces of fixture equipment and stretched by a manually tuned axis of rotation. The screw was used to fix the PDMS fiber again. A white light source (Hamamatsu, L10290) was employed to illuminate the PDMS fiber. The transmission spectrum was recorded by using a spectrometer (Ocean Optics, USB4000, 200–1100 nm).

**Figure 5:**
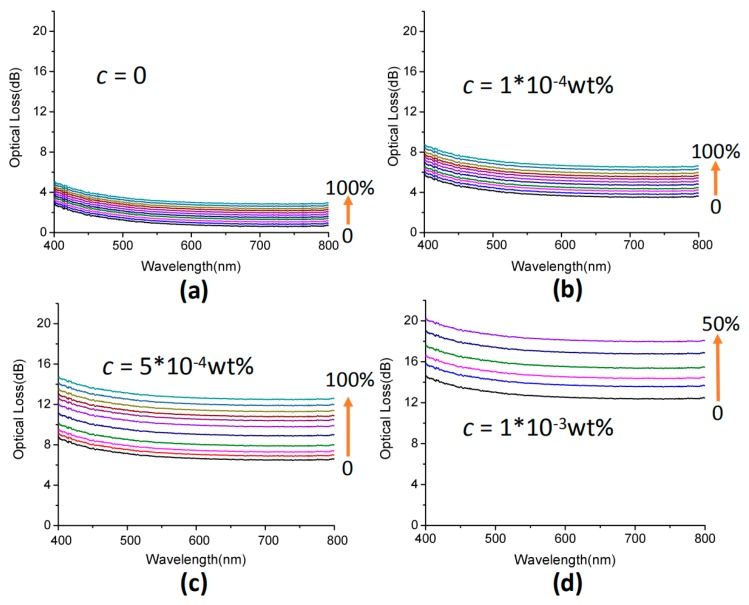
(**a**–**d**) Attenuation spectra of the PDMS fiber with different concentrations of graphene in stretching states. The strain started at ε=0 and increased by 10% per step.

Accompanied with the action of strain, the optical loss changed, as shown in Figure 5. In the experiment, we used the change of length to reflect the magnitude of the stress. The original length of the sample tested was 3 cm, as seen in Figure 5, the elongation of each step was 3 mm, and the length was 6 cm when the sample reached 100% strain. In other words, the stress in Figure 5 increased by 10% per step. As can be seen from Figure 5a, PDMS fiber without graphene had a limited absorption amount and attenuation had no obvious changes under the tensile state. When adding graphene to PDMS fiber, it was obvious that the attenuation changed in light intensity under the tensile state, as shown in Figure 5b–d. Compared with Figure 5a, when the concentration of graphene was $5\times 10^{-4}\;\mathrm{wt}\;\%$., the optical loss of PDMS fiber was four times under the maximum tensile state, and the sensitivity was significantly improved, which conforms to the strain sensing mechanism. When the concentration of graphene was too high, the light intensity could not be detected when the tensile strain reached a certain level, and the signal-to-noise ratio worsened, as shown in Figure 5d. Therefore, we chose a sensor with a graphene concentration of $5\times 10^{-4}\;\mathrm{wt}\;\%$.

**Figure 6:**
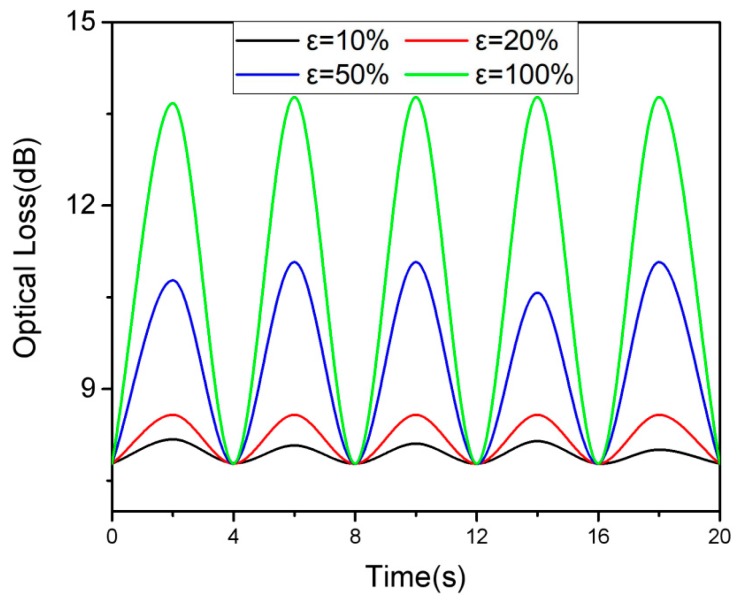
Under different loading strains, dynamic responses of the PDMS fiber to repeated. Strains of 10%, 20%, 50%, and 100%.

In order to study the dynamic response of the sensor during stretching and releasing, we performed a cyclic test on a sensor with a graphene concentration of $5\times 10^{-4}\;\mathrm{wt}\;\%$ to study its actual sensing capability through repeated measurements. Figure 6 shows the changes in the sensor’s tensile/release for five cycles under different stresses. The linear response between strain and optical loss is also illustrated in Figure 6.

### 3.3. Motion-Detecting Applications

**Figure 7:**
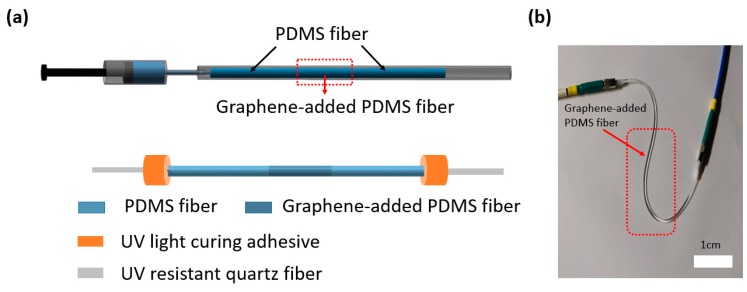
(**a**) Schematic showing the preparation and fixation of the sensor. (**b**) Digital optical image of fiber sensor.

Due to its high flexibility, sensitivity and large strain range, the sensor can be directly attached to the human body to monitor its movement in real time. Because the graphene material has no wavelength selectivity in a visible light band, we can measure the optical loss through PDMS fibers with a single wavelength laser. Combined with the practical application of the sensor, we have improved the process of the optical fiber as shown in Figure 7a. We first filled PDMS into the tube mold, then injected graphene-added PDMS into the tube mold, with a fixed length of 3 cm. Finally, we injected PDMS into the tube mold and got the optical fiber sensor after curing. The sensor was pigtailed with two UV resistant quartz fibers (core: 600 μm) at each end and encapsulated with UV light curing adhesive. The practical application of optical fiber sensing is shown in Figure 7b.

**Figure 8:**
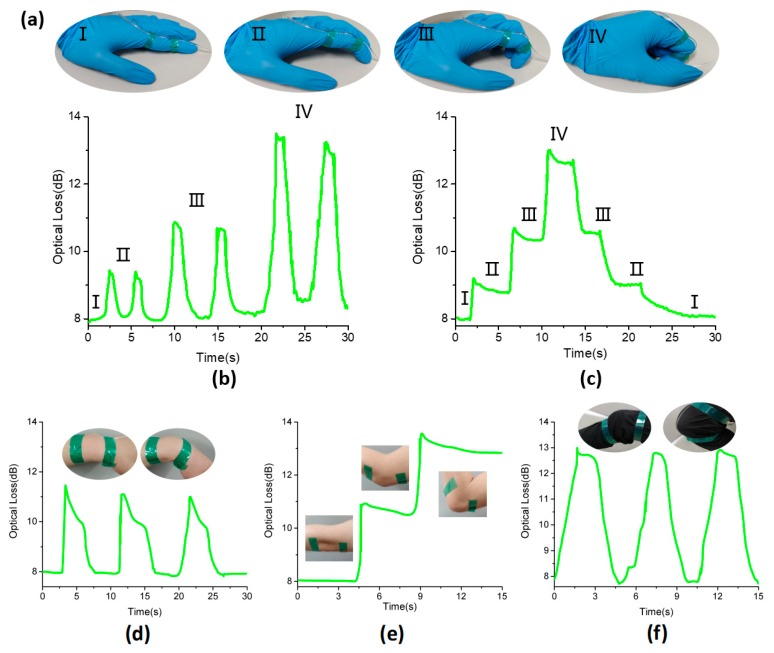
(**a**) Sensors on the fingers, from gradually bending to relaxing. (**b**) Changes in optical loss of index finger bending motion at different bending angles. (**c**) The change in optical loss of the sensor corresponded to the state of (a). (**d**) The variation of optical loss caused by wrist bending. (**e**) The variation of optical loss caused by elbow bending. (**f**) Changes in optical loss due to knee bending.

For large-strain detection of human motion, we attached sensors to human joints, as shown in Figure 8. Figure 8a shows the change in optical loss intensity caused by the index finger bending. When the finger bent at a certain angle, the optical loss intensity of the strain sensor rapidly increased to a certain value. In addition, a gradual response was observed when the fingers were bent. The larger the bending angle, the higher the increase of the optical loss intensity, as shown in Figure 8b. Figure 8c shows the optical loss change of the index finger from bending to releasing; when the finger returned to its original position, the optical loss intensity returned. We also attached fiber optic sensors to the wrist joints, as shown in Figure 8d, to detect wrist motion. The sensor can easily detect the stretching and bending of the wrist. The optical loss changes of the wrist over several bending cycles demonstrated a high degree of reproducibility. The proposed sensor also detects elbow movement. Moreover, testing was conducted during performance of a stepped bending sequence in which the elbow was bent and held for a few seconds at each position. Figure 8e shows the variation of optical loss of the sensor at different bending stages over time. It can be seen that the optical loss of the sensor increased as the bending angle increased. Furthermore, when the sensor was used to further monitor other larger types of human motion, such as knee bending, stable and repeatable responses were also observed, as shown in Figure 8f. These results indicate that the proposed flexible and optical fiber sensors could be used for identifying different bending angles of joints, which can detect and quantify human joint bending.

**Figure 9:**
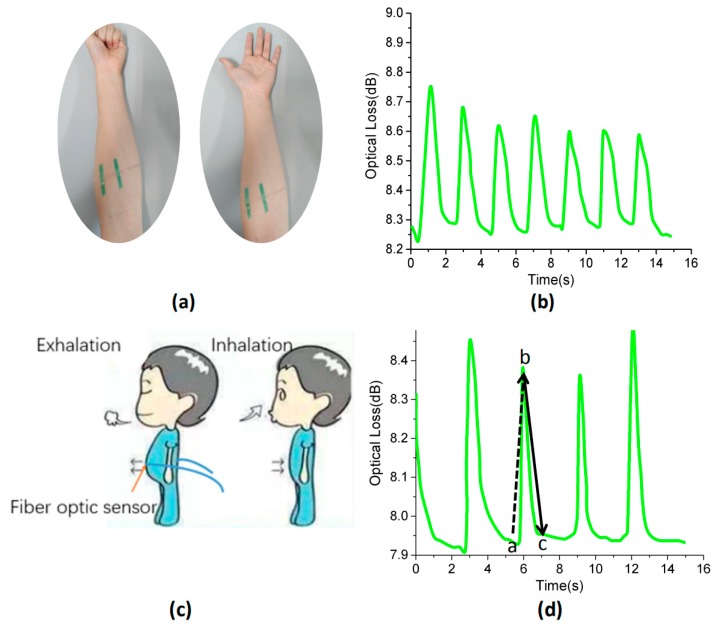
(**a**) Photograph of the fiber optic sensor attached to the wrist. (**b**) Optical loss of the fiber optic sensor over time during the forearm muscle movement. (**c**) Schematic illustration showing a breath sensor fabricated by attaching a fiber optic sensor directly above the abdomen. (**d**) Optical loss of the fiber optic sensor over time during human breath monitoring. Among them, (a,b) are exhalation, (b,c) are inhalation.

In addition, we also did some tests for smaller strains such as muscle movements, as shown in Figure 9. When the hand is clenched and the fingers are relaxed, the muscle relaxation of the arm is different. Figure 9b shows the changes of optical loss caused by the state of the muscles when the forearms were tensed and relaxed. Moreover, the sensors were installed on the belly of a person, as shown in Figure 9c. The upward section in the sensor output signal represents exhalation, and the downward section of the output curve represents inhalation. As shown in Figure 9d, the sensor generated a repeatable and regular signal pattern during respiration, with a respiration rate of 20 breaths/min ^−1^.

**Figure 10:**
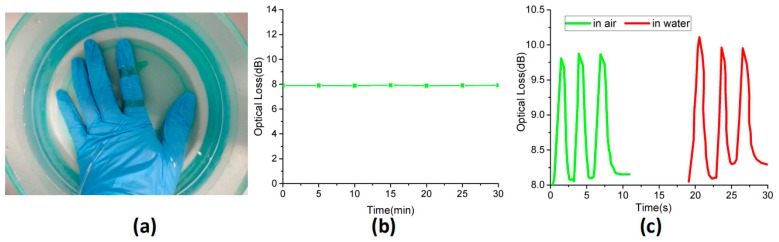
(**a**) Diagram of a sensor mounted on the index finger in a water environment. (**b**) The optical loss of sensor varied with the insulation time. (**c**) Dynamic response of finger bending in different environments.

The stability of the sensor is necessary for practical applications. We soaked the sensor in water to monitor its optical response to water. Figure 10b shows the optical loss of the sensor with the insulation time. From this diagram, it can be pointed out that there was no significant change in the light intensity loss after 30 min of immersion in water. In addition, the sensor was attached to the back of the index finger to bond the two ends of the telescopic tape, and the sensor’s dynamic response in water was recorded at room temperature. Because of the sensor’s flexibility, it could achieve adhesion to uneven skin. Figure 10c shows the change in light intensity loss of the sensor, when the finger bent in air and water at room temperature. Figure 10d clearly demonstrates that the dynamic responses of the sensor in air and water were similar, indicating that the sensor had good waterproof performance. This may be due to the fact that the fiber optic sensor was completely sealed with hydrophobic silicone rubber, preventing water molecules from being absorbed into the sensor surface. Therefore, the influence of water on a sensor can be effectively reduced.

## 4. Conclusions

In conclusion, we have demonstrated a simple, efficient and low-cost way to manufacture strain sensors with high tensile properties and sensitivity, made from graphene and polymer elastomers. Compared to the pure PDMS fiber, the composite fiber significantly enhances the optical sensing signal of the sensor, showing excellent sensitivity in the case of strain sensing. The tensile property of the sensor is 150%. This strain sensor was applied to practical applications to detect finger bending, wrist bending, elbow bending, knee bending and other motions of the human body in an all-round way. In addition, the optic fiber sensors could be used to monitor muscle movements and breathing. Moreover, we also researched the sensing capacity (bending strain) of this fiber sensor in water, and found that it has certain waterproof function. This study shows that the sensor fibers have a wide range of potential applications in flexible and stretchable devices.
